# The genome sequence of the bluish flesh fly, *Sarcophaga  *( *Robineauella*) *caerulescens* (Zetterstedt, 1838)

**DOI:** 10.12688/wellcomeopenres.18718.1

**Published:** 2023-01-12

**Authors:** Steven Falk, University of Oxford and Wytham Woods Genome Acquisition Lab, Darwin Tree of Life Barcoding collective, Wellcome Sanger Institute Tree of Life programme, Wellcome Sanger Institute Scientific Operations: DNA Pipelines collective, Tree of Life Core Informatics collective, John F. Mulley, Darwin Tree of Life Consortium

**Affiliations:** 1 Independent researcher, Kenilworth, Warwickshire,, UK; 2 School of Natural Sciences, Bangor University, Bangor, Wales, UK

**Keywords:** Sarcophaga caerulescens, bluish flesh fly, genome sequence, chromosomal, Diptera

## Abstract

We present a genome assembly from an individual male *Sarcophaga caerulescens* (the bluish flesh fly; Arthropoda; Insecta; Diptera; Sarcophagidae). The genome sequence is 597 megabases in span. Most of the assembly is scaffolded into seven chromosomal pseudomolecules, including the assembled X and Y sex chromosomes. The mitochondrial genome has also been assembled and is 21.1 kilobases in length. Gene annotation of this assembly on Ensembl identified 16,559 protein coding genes.

## Species taxonomy

Eukaryota; Metazoa; Ecdysozoa; Arthropoda; Hexapoda; Insecta; Pterygota; Neoptera; Endopterygota; Diptera; Brachycera; Muscomorpha; Oestroidea; Sarcophagidae; Sarcophaga; *Robineauella*; *Sarcophaga caerulescens* (Zetterstedt, 1838) (NCBI:txid596942).

## Background

*Sarcophaga caerulescens* (Diptera: Sarcophagidae) is a widely distributed flesh fly reported from Nearctic, Palearctic and Oriental regions ( Pape, 1996). The species name reflects its bluish colouration (indeed it is sometimes referred to as the bluish flesh fly), and individuals possess the characteristic pigmentation pattern of the genus, with three longitudinal stripes on the thorax and a checked pattern on the abdomen. There are roughly 890 species in the genus *Sarcophaga*, divided into approximately 169 subgenera ( Buenaventura *et al.*, 2017), and *S. caerulescens* is in the *Robineauella* subgenus along with around 15 other species, a striking contrast to most other Sarcophagid subgenera, which are monotypic.

Data regarding distribution and abundance of *S. caerulescens*, as with most, if not all, of the 65 currently recognised UK Sarcophagid species, is relatively sparse, most likely as a result of the difficulty in differentiating highly similar species visually or from photographs. Available data from the National Biodiversity Atlas (NBN) Atlas ( https://species.nbnatlas.org/species/NBNSYS0100005387) shows highest abundance in summer months (June–August), with multiple reports from England and Wales, and a single record from Scotland. As with many flesh fly species, *S. caerulescens* has been recorded in association with human and animal carcasses, and has been suggested as a candidate for forensic entomology and determination of post-mortem interval (PMI). Carcass colonisation has been reported from both open and rural habitats ( Fremdt & Amendt, 2014; Ren *et al.*, 2018; Szpila *et al.*, 2015), and indoors ( Pohjoismäki *et al.*, 2010), and habitat preferences likely vary regionally. All members of the Sarcophagidea examined to date have a diploid chromosome number of 12, with an XY sex determination system, where males are the heterogametic sex ( Srivastava & Gaur, 2015).

The *S. caerulescens* genome should be a useful resource for the development of improved DNA barcoding markers, where current COI-based assays can struggle to differentiate some species ( Jordaens *et al.*, 2013), and provide useful insights into the evolution of ovoviviparity.

### Genome sequence report

**Figure 1.**
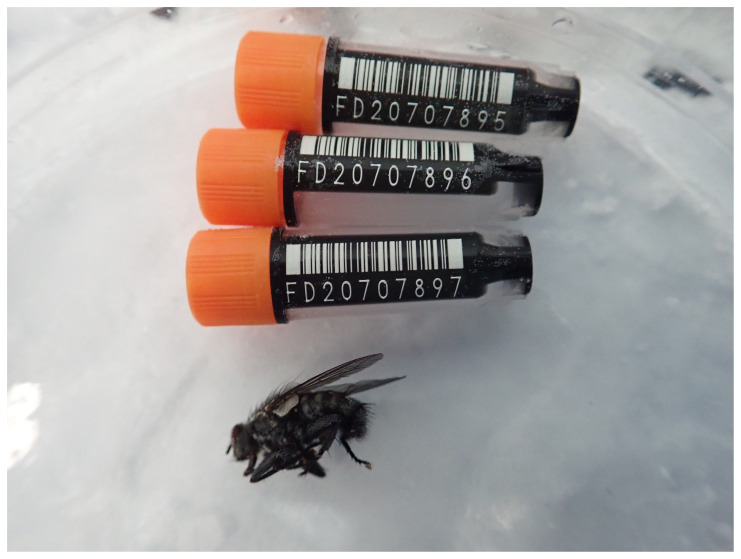
Image of the *Sarcophaga caerulescens* (idSarCaer1) specimen used for genome sequencing.

The genome was sequenced from one male *Sarcophaga caerulescens* ( Figure 1) collected from Wytham Woods, Berkshire (latitude 51.77, longitude –1.33). A total of 25-fold coverage in Pacific Biosciences single-molecule HiFi long reads and 57-fold coverage in 10X Genomics read clouds were generated. Primary assembly contigs were scaffolded with chromosome conformation Hi-C data. Manual assembly curation corrected 235 missing or mis-joins and removed 10 haplotypic duplications, reducing the assembly length by 0.61% and the scaffold number by 41.03%, and increasing the scaffold N50 by 69.4%.

**Figure 2.**
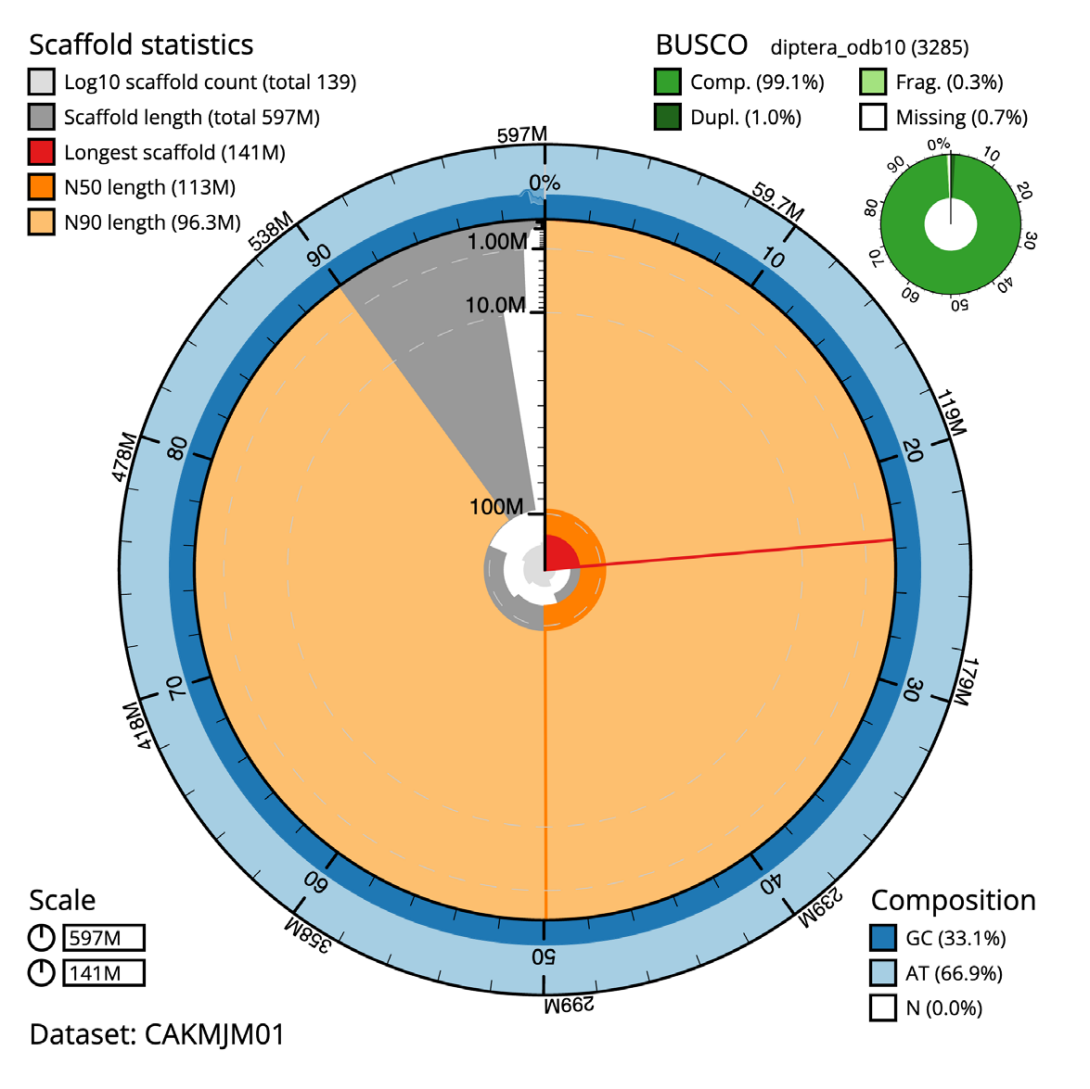
Genome assembly of *Sarcophaga caerulescens*, idSarCaer1.1: metrics. The BlobToolKit Snailplot shows N50 metrics and BUSCO gene completeness. The main plot is divided into 1,000 size-ordered bins around the circumference with each bin representing 0.1% of the 597,323,580 bp assembly. The distribution of chromosome lengths is shown in dark grey with the plot radius scaled to the longest chromosome present in the assembly (141,445,521 bp, shown in red). Orange and pale-orange arcs show the N50 and N90 chromosome lengths (113,339,294 and 96,303,733 bp), respectively. The pale grey spiral shows the cumulative chromosome count on a log scale with white scale lines showing successive orders of magnitude. The blue and pale-blue area around the outside of the plot shows the distribution of GC, AT and N percentages in the same bins as the inner plot. A summary of complete, fragmented, duplicated and missing BUSCO genes in the diptera_odb10 set is shown in the top right. An interactive version of this figure is available at https://blobtoolkit.genomehubs.org/view/idSarCaer1.1/dataset/CAKMJM01/snail.

The final assembly has a total length of 597 Mb in 138 sequence scaffolds with a scaffold N50 of 113.3 Mb ( Table 1). Most (98.99%) of the assembly sequence was assigned to seven chromosomal-level scaffolds, representing five autosomes and the X and Y sex chromosomes. Chromosome-scale scaffolds are named by synteny based on the assembly for *Sarcophaga peregrina* (GCA_014635995.1) ( Figure 2– Figure 5; Table 2). The assembly has a BUSCO v5.3.2 ( Manni *et al.*, 2021) completeness of 99.1% using the diptera_odb10 reference set. While not fully phased, the assembly deposited is of one haplotype. Contigs corresponding to the second haplotype have also been deposited.

**Table 1.** Genome data for *Sarcophaga caerulescens*, idSarCaer1.1.

Project accession data		
Assembly identifier	idSarCaer1.1	
Species	*Sarcophaga caerulescens*	
Specimen	idSarCaer1	
NCBI taxonomy ID	596942	
BioProject	PRJEB48188	
BioSample ID	SAMEA7746589	
Isolate information		
Assembly metrics *		*Benchmark*
Consensus quality (QV)	54.2	*≥ 50*
*k*-mer completeness	99.99%	*≥ 95%*
BUSCO **	C:99.1%[S:98.0%,D:1.0%], F:0.3%,M:0.7%,n:3,285	*C ≥ 95%*
Percentage of assembly mapped to chromosomes	98.99%	*≥ 95%*
Sex chromosomes	X and Y	*localised homologous pairs*
Organelles	Mitochondrial genome assembled	*complete single alleles*
Raw data accessions		
PacificBiosciences SEQUEL II	ERR7169027	
10X Genomics Illumina	ERR7167665–ERR7167668	
Hi-C Illumina	ERR7167664	
PolyA RNA-Seq Illumina	ERR10123661	
Genome assembly		
Assembly accession	GCA_927399465.1	
*Accession of alternate haplotype*	GCA_927399425.1	
Span (Mb)	597.3	
Number of contigs	595	
Contig N50 length (Mb)	3.0	
Number of scaffolds	138	
Scaffold N50 length (Mb)	113.3	
Longest scaffold (Mb)	141.45	
Genome annotation		
Number of protein-coding genes	16,559	

* Assembly metric benchmarks are adapted from column VGP-2020 of “Table 1: Proposed standards and metrics for defining genome assembly quality” from ( Rhie *et al.*, 2021).

** BUSCO scores based on the diptera_odb10 BUSCO set using v5.3.2. C = complete [S = single copy, D = duplicated], F = fragmented, M = missing, n = number of orthologues in comparison. A full set of BUSCO scores is available at https://blobtoolkit.genomehubs.org/view/idSarCaer1.1/dataset/CAKMJM01/busco.

**Figure 3.**
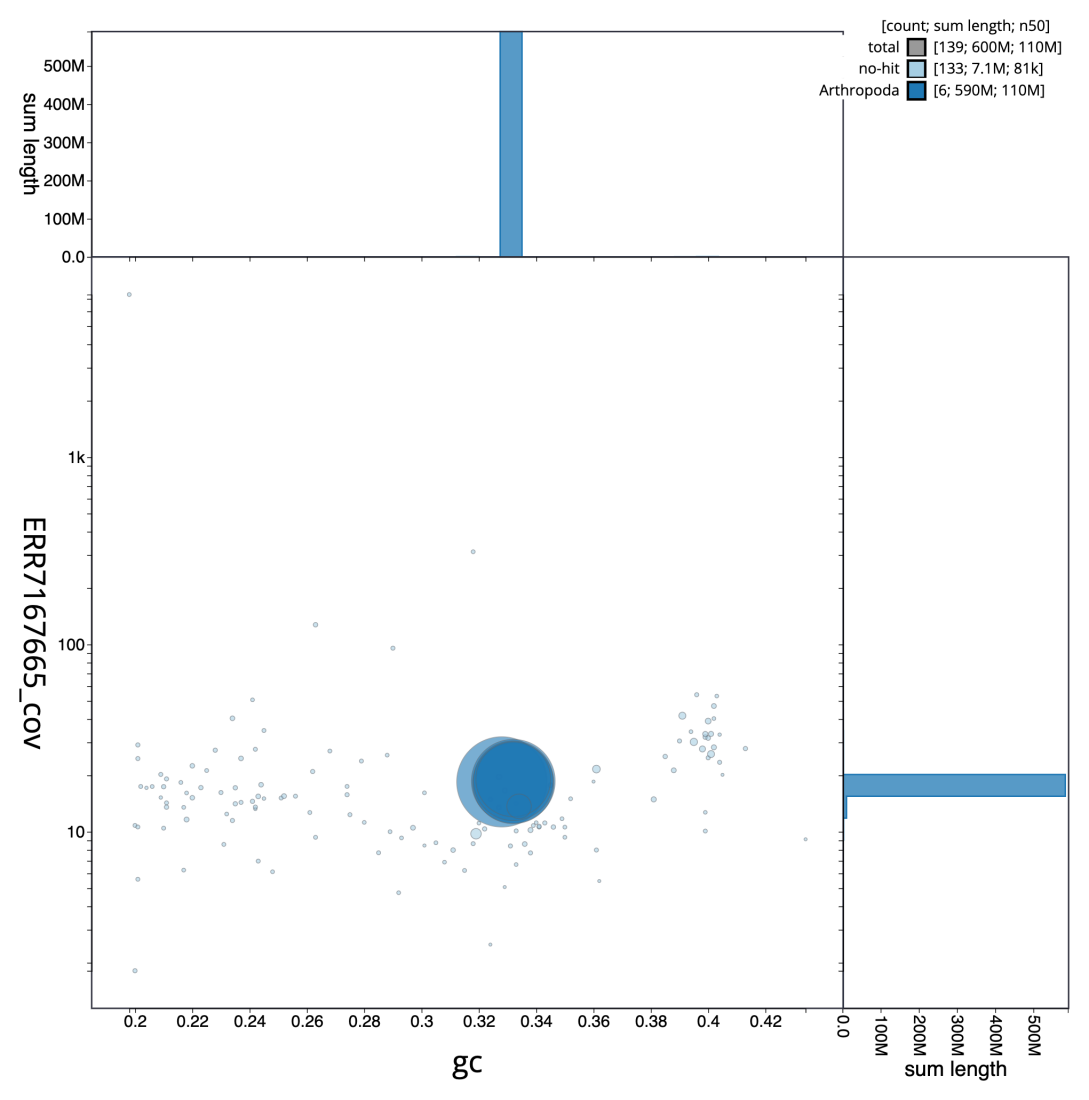
Genome assembly of *Sarcophaga caerulescens*, idSarCaer1.1: GC coverage. BlobToolKit GC-coverage plot. Scaffolds are coloured by phylum. Circles are sized in proportion to scaffold length. Histograms show the distribution of chromosome length sum along each axis. An interactive version of this figure is available at https://blobtoolkit.genomehubs.org/view/idSarCaer1.1/dataset/CAKMJM01/blob.

**Figure 4.**
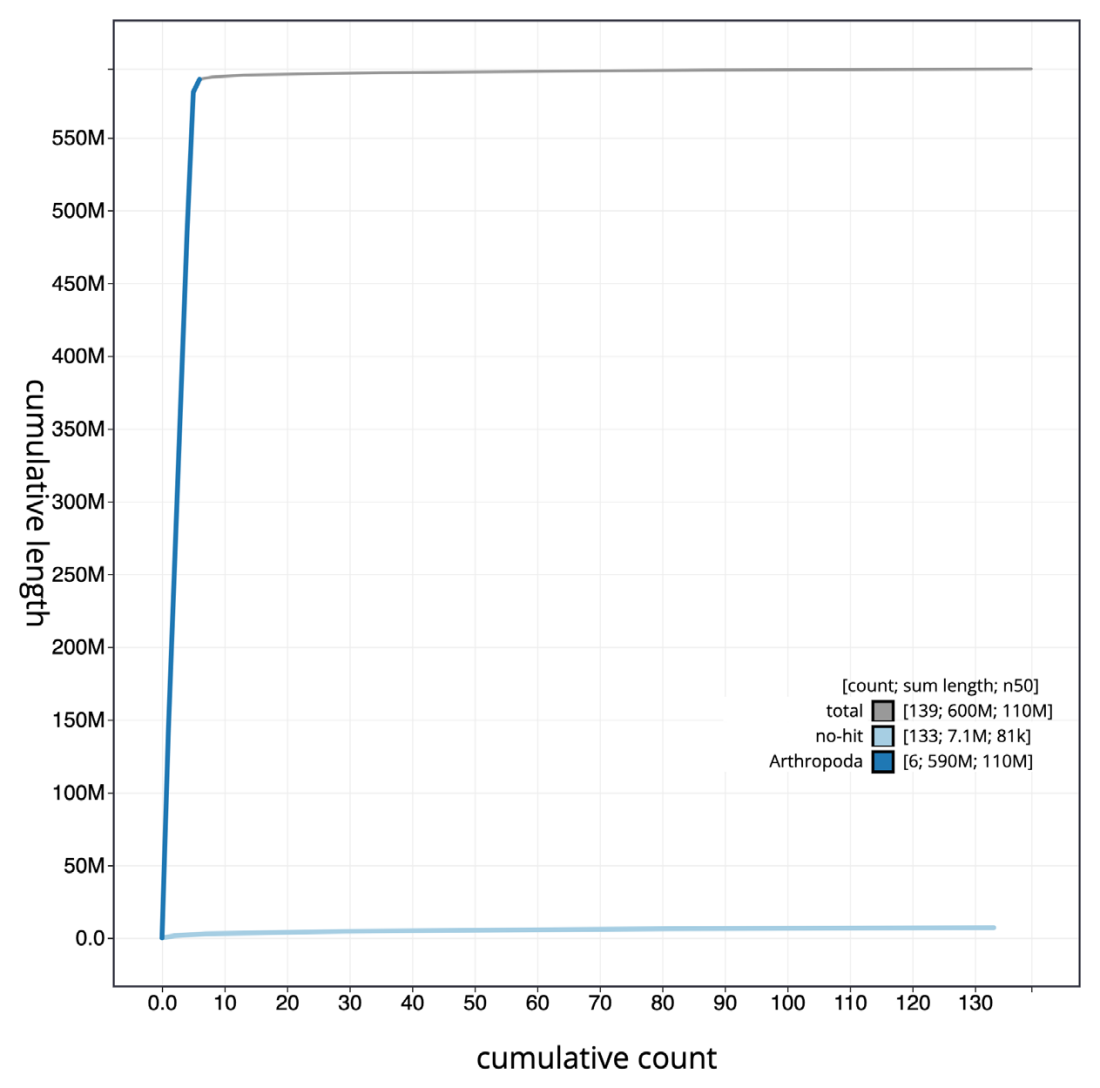
Genome assembly of *Sarcophaga caerulescens*, idSarCaer1.1: cumulative sequence. BlobToolKit cumulative sequence plot. The grey line shows cumulative length for all scaffolds. Coloured lines show cumulative lengths of scaffolds assigned to each phylum using the buscogenes taxrule. An interactive version of this figure is available at https://blobtoolkit.genomehubs.org/view/idSarCaer1.1/dataset/CAKMJM01/cumulative.

**Figure 5.**
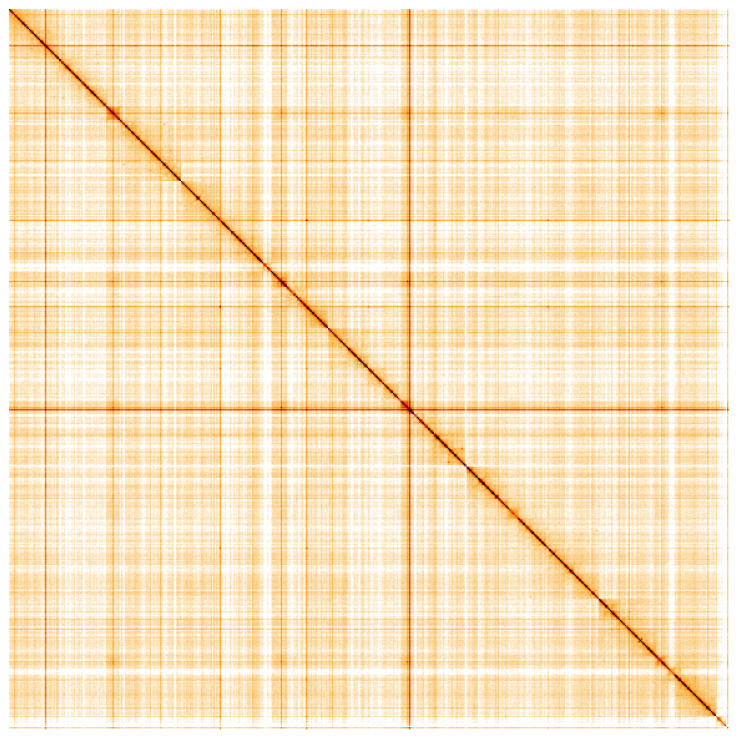
Genome assembly of *Sarcophaga caerulescens*, idSarCaer1.1: Hi-C contact map. Hi-C contact map of the idSarCaer1.1 assembly, visualised using HiGlass. Chromosomes are shown in order of size from left to right and top to bottom. An interactive version of this figure may be viewed at https://genome-note-higlass.tol.sanger.ac.uk/l/?d=YnFQ9N3hSom1qDHlV87b0A.

**Table 2.** Chromosomal pseudomolecules in the genome assembly of *Sarcophaga caerulescens*, idSarCaer1.

INSDC accession	Chromosome	Size (Mb)	GC%
OV656872.1	1	141.45	32.8
OV656873.1	2	121.02	33.2
OV656866.1	3	113.34	33.2
OV656867.1	4	109.34	33.2
OV656868.1	5	96.3	33.2
OV656869.1	X	8.75	33.4
OV656870.1	Y	1.1	31.9
OV656871.1	MT	0.02	20.1
-	unplaced	6	32.7

### Genome annotation report

The GCA_927399465.1 genome was annotated using the Ensembl rapid annotation pipeline ( Table 1; https://rapid.ensembl.org/Sarcophaga_caerulescens_GCA_927399465.1/). The resulting annotation includes 40,319 transcribed mRNAs from 16,559 protein-coding and 12,343 non-coding genes.

## Methods

### Sample acquisition and nucleic acid extraction

A male *S. caerulescens* (idSarCaer1) was collected using a net in Wytham Woods, Berkshire, UK (latitude 51.77, longitude –1.33) by Steven Falk (independent researcher). The specimen was identified by Steven Falk and snap-frozen on dry ice.

DNA was extracted at the Tree of Life laboratory, Wellcome Sanger Institute (WSI). The idSarCaer1 sample was weighed and dissected on dry ice with head tissue set aside for Hi-C sequencing. Thorax tissue was disrupted using a Nippi Powermasher fitted with a BioMasher pestle. High molecular weight (HMW) DNA was extracted using the Qiagen MagAttract HMW DNA extraction kit. Low molecular weight DNA was removed from a 20 ng aliquot of extracted DNA using 0.8X AMpure XP purification kit prior to 10X Chromium sequencing; a minimum of 50 ng DNA was submitted for 10X sequencing. HMW DNA was sheared into an average fragment size of 12–20 kb in a Megaruptor 3 system with speed setting 30. Sheared DNA was purified by solid-phase reversible immobilisation using AMPure PB beads with a 1.8X ratio of beads to sample to remove the shorter fragments and concentrate the DNA sample. The concentration of the sheared and purified DNA was assessed using a Nanodrop spectrophotometer and Qubit Fluorometer and Qubit dsDNA High Sensitivity Assay kit. Fragment size distribution was evaluated by running the sample on the FemtoPulse system.

RNA was extracted from abdomen tissue of idSarCaer1 in the Tree of Life Laboratory at the WSI using TRIzol, according to the manufacturer’s instructions. RNA was then eluted in 50 μl RNAse-free water and its concentration assessed using a Nanodrop spectrophotometer and Qubit Fluorometer using the Qubit RNA Broad-Range (BR) Assay kit. Analysis of the integrity of the RNA was done using Agilent RNA 6000 Pico Kit and Eukaryotic Total RNA assay.

### Sequencing

Pacific Biosciences HiFi circular consensus and 10X Genomics read cloud DNA sequencing libraries were constructed according to the manufacturers’ instructions. Poly(A) RNA-Seq libraries were constructed using the NEB Ultra II RNA Library Prep kit. DNA and RNA sequencing was performed by the Scientific Operations core at the WSI on Pacific Biosciences SEQUEL II (HiFi), Illumina NovaSeq 6000 (RNA-Seq and 10X) instruments. Hi-C data were also generated from head tissue of idSarCaer1 using the Arima v2 kit and sequenced on the NovaSeq 6000 instrument.

### Genome assembly

Assembly was carried out with Hifiasm ( Cheng *et al.*, 2021) and haplotypic duplication was identified and removed with purge_dups ( Guan *et al.*, 2020). One round of polishing was performed by aligning 10X Genomics read data to the assembly with Long Ranger ALIGN, calling variants with freebayes ( Garrison & Marth, 2012). The assembly was then scaffolded with Hi-C data ( Rao *et al.*, 2014) using YaHS ( Zhou *et al.*, 2022). The assembly was checked for contamination and corrected using the gEVAL system ( Chow *et al.*, 2016) as described previously ( Howe *et al.*, 2021). Manual curation was performed using gEVAL, HiGlass ( Kerpedjiev *et al.*, 2018) and Pretext ( Harry, 2022). The mitochondrial genome was assembled using MitoHiFi ( Uliano-Silva *et al.*, 2021), which performed annotation using MitoFinder ( Allio *et al.*, 2020). The genome was analysed and BUSCO scores generated within the BlobToolKit environment ( Challis *et al.*, 2020). Table 3 contains a list of all software tool versions used, where appropriate.

**Table 3.** Software tools and versions used.

Software tool	Version	Source
BlobToolKit	3.4.0	Challis *et al.*, 2020
freebayes	1.3.1-17-gaa2ace8	Garrison & Marth, 2012
gEVAL	N/A	Chow *et al.*, 2016
Hifiasm	0.15.3	Cheng *et al.*, 2021
HiGlass	1.11.6	Kerpedjiev *et al.*, 2018
Long Ranger ALIGN	2.2.2	https://support.10xgenomics.com/genome-exome/software/ pipelines/latest/advanced/other-pipelines
MitoHiFi	2	Uliano-Silva *et al.*, 2021
PretextView	0.2	Harry, 2022
purge_dups	1.2.3	Guan *et al.*, 2020
YaHS	1.0	Zhou *et al.*, 2022

### Genome annotation

The Ensembl gene annotation system ( Aken *et al.*, 2016) was used to generate annotation for the *Sarcophaga caerulescens* assembly (GCA_927399465.1). Annotation was created primarily through alignment of transcriptomic data to the genome, with gap filling via protein to-genome alignments of a select set of proteins from UniProt ( UniProt Consortium, 2019).

### Ethics/compliance issues

The materials that have contributed to this genome note have been supplied by a Darwin Tree of Life Partner. The submission of materials by a Darwin Tree of Life Partner is subject to the Darwin Tree of Life Project Sampling Code of Practice. By agreeing with and signing up to the Sampling Code of Practice, the Darwin Tree of Life Partner agrees they will meet the legal and ethical requirements and standards set out within this document in respect of all samples acquired for, and supplied to, the Darwin Tree of Life Project. Each transfer of samples is further undertaken according to a Research Collaboration Agreement or Material Transfer Agreement entered into by the Darwin Tree of Life Partner, Genome Research Limited (operating as the Wellcome Sanger Institute), and in some circumstances other Darwin Tree of Life collaborators.

## Data Availability

European Nucleotide Archive: *Sarcophaga caerulescens*. Accession number PRJEB48188; https://identifiers.org/ena.embl/PRJEB48188 ( Wellcome Sanger Institute, 2022). The genome sequence is released openly for reuse. The *Sarcophaga caerulescens* genome sequencing initiative is part of the Darwin Tree of Life (DToL) project. All raw sequence data and the assembly have been deposited in INSDC databases. Raw data and assembly accession identifiers are reported in Table 1. Members of the University of Oxford and Wytham Woods Genome Acquisition Lab are listed here: https://doi.org/10.5281/zenodo.4789928. Members of the Darwin Tree of Life Barcoding collective are listed here: https://doi.org/10.5281/zenodo.4893703. Members of the Wellcome Sanger Institute Tree of Life programme are listed here: https://doi.org/10.5281/zenodo.4783585. Members of Wellcome Sanger Institute Scientific Operations: DNA Pipelines collective are listed here: https://doi.org/10.5281/zenodo.4790455. Members of the Tree of Life Core Informatics collective are listed here: https://doi.org/10.5281/zenodo.5013541. Members of the Darwin Tree of Life Consortium are listed here: https://doi.org/10.5281/zenodo.4783558.
