# Supplementary material for: The genome sequence of the bluish flesh fly, Sarcophaga  ( Robineauella) caerulescens (Zetterstedt, 1838)
Source: Wellcome Open Res. 2023 Jan 12;8:17. [Version 1] doi: 10.12688/wellcomeopenres.18718.1 (PMC10288162; doi:10.12688/wellcomeopenres.18718.1)
Supplement: Peer review history

# Peer review: Reviewer response for version 1

**DOI:** 10.21956/wellcomeopenres.20756.r60062

**Published:** 2023-06-26

**Authors:** Andrzej Grzywacz

**Affiliations:** 1 Nicolaus Copernicus University, Toruń, Poland

The report of *Sarcophaga  *( *Robineauella*) *caerulescens *genome is satisfactory and contains all the necessary information. Introduction covers most important aspects of *Sarcophaga caerulescens* life history with the most recent references. The genome assembly is of high quality. A brief explanation of larval predatory behaviour and ovoviviparity in *Sarcophaga caerulescens* will highlight why it is an interesting model for comparative genomics.

 I recommend adding more information concerning specimen identification. In particular, a reference to a taxonomic key used for species delimitation, i.e., latest species-level taxonomic literature that contains the currently accepted species concept ^1^.

Are sufficient details of methods and materials provided to allow replication by others?

Partly

Is the rationale for creating the dataset(s) clearly described?

Yes

Are the datasets clearly presented in a useable and accessible format?

Yes

Are the protocols appropriate and is the work technically sound?

Yes

Reviewer Expertise:

Systematics, entomology, genomics

I confirm that I have read this submission and believe that I have an appropriate level of expertise to confirm that it is of an acceptable scientific standard.

## References

[1] : Citation of taxonomic publications: the why, when, what and what not. *Systematic Entomology* .2017;42(2) : 10.1111/syen.12215 301-304 10.1111/syen.12215

# Peer review: Reviewer response for version 1

**DOI:** 10.21956/wellcomeopenres.20756.r58227

**Published:** 2023-06-22

**Authors:** John Soghigian

**Affiliations:** 1 University of Calgary, Calgary, Alberta, Canada

Falk et al. is a report on the genome sequence of *Sarcophaga caerulescens*, a flesh fly that is occasionally found throughout temperate North America and Europe. The report provides ample details on the methods used to acquire samples, the sequencing technologies used, and the bioinformatics applications relied upon to assemble, scaffold, and annotate the genome. The genome is of similar high quality to other recent reference genome sequencing projects, and will provide for useful comparative genomics in the future. 

 I have no serious concerns, but below are quite minor comments that the authors may wish to consider:

- The ovoviviparity of the Sarcophagidae deserves a mention in the introduction.
- While the genome will certainly help with barcoding, I think it has a greater potential in comparative genomics of flies. This is a high quality genome that will certainly provide significant insight into the radiation of the Oestroidea and their unique adaptations to different substrates for larval development.

Are sufficient details of methods and materials provided to allow replication by others?

Yes

Is the rationale for creating the dataset(s) clearly described?

Yes

Are the datasets clearly presented in a useable and accessible format?

Yes

Are the protocols appropriate and is the work technically sound?

Yes

Reviewer Expertise:

Systematics, genomics

I confirm that I have read this submission and believe that I have an appropriate level of expertise to confirm that it is of an acceptable scientific standard.

# Peer review: Reviewer response for version 1

**DOI:** 10.21956/wellcomeopenres.20756.r58223

**Published:** 2023-06-02

**Authors:** Nicolas Pollet

**Affiliations:** 1 EGCE, CNRS, Universite Paris-Saclay, Gif-sur-Yvette, Île-de-France, France

This genome report for the bluish flesh fly, *Sarcophaga  *( *Robineauella*) *caerulescens* (Zetterstedt, 1838) is rather complete, the genome characteristics are sound for a dipteran. Regarding sample acquisition and nucleic acid extraction, I think it would be beneficial to the community to explicitly provide either the range of acceptable values for spectrophotometric, concentration measures, and femtopulse data or to give the values obtained. Many thanks to all who participated.

Are sufficient details of methods and materials provided to allow replication by others?

Partly

Is the rationale for creating the dataset(s) clearly described?

Yes

Are the datasets clearly presented in a useable and accessible format?

Yes

Are the protocols appropriate and is the work technically sound?

Yes

Reviewer Expertise:

Genomics and evolution

I confirm that I have read this submission and believe that I have an appropriate level of expertise to confirm that it is of an acceptable scientific standard.
